# Phytochemical Analysis of the Essential Oil from Botanically Certified Oleogum Resin of *Boswellia sacra* (Omani Luban)

**DOI:** 10.3390/molecules13092181

**Published:** 2008-09-16

**Authors:** Ahmed Al-Harrasi, Salim Al-Saidi

**Affiliations:** Department of Chemistry, College of Science, Sultan Qaboos University, P.O Box 36, Al-Khod 123, Oman; E-mail: salsaidi@squ.edu.om (S. A-S.)

**Keywords:** Essential oil, *Boswellia sacra*, Monoterpenes, Sesquiterpenes

## Abstract

The yield of hydrodistillation of a botanically certified Oleogum Resin of Boswellia sacra essential oil (5.5%); and its chemical constituents were determined. The GC/MS technique was used for the analysis of the oil. Several oil components were identified based upon comparison of their mass spectral data with those of reference compounds published in literature or stored in a computer library. The oil was characterized by the high content of the monoterpenes (34) which constituted 97.3% in which *E*-β-ocimene and limonene were the major constituents. The remaining 2.7% was accounted for the sesquiterpenes (16) in which the *E*-caryophyllene was the major constituent. The analysis proved the complete absence of the diterpenes.

## Introduction

Frankincense or olibanum is a plant product. It is an oleo-gum-resin produced by several species of tree belonging to the *Boswellia* genus and the family Burseraceae, which is characterized by resin bearing ducts. There are some 15 members of this much revised genus. The trees are short, with papery bark, star-like flowers and compound leaves [1, 2]. *Boswellia sacra* is a tree indigenous to the Dhofar region of the Sultanate of Oman. *Boswellia sacra* trees are up to five meters tall, either with a single or several trunks rising from the base, papery, peeling bark and densely tangled branches with leaves clustered at the ends. 

The constituents of the essential oil of frankincense were first investigated by Stenhouse in 1840, when he identified fourteen monoterpenoic constituents, depending on the origin of the resin. These included pinene, dipentene, phellandrene and cadinene [3]. The acidic constituent of olibaum, boswellic acid, with a molecular formula of C_32_H_52_O_4_ was first reported by Tschirch and Halbey in 1898. The exact structure could not be established at that time [4]. A detailed investigation of the olibanum resin was performed by Winterstein and Stein in 1932 [5]. Later in the 1960s, several boswellic acids such as α- and β-boswellic acids, 11α-hydroxy-β-boswellic acid and 3-*O*-acetyl-11-hydroxy-β-boswellic acid were identified by various derivatisation methods. In 1967, Snatzke and Vértesy reported the structures of acetyl-11-keto-β-boswellic acid [6]. In 1978 Pardhy and Bhattacharya identified tirucallic acids as well as β-boswellic acid, acetyl-β-boswellic acid, 11-keto-β-boswellic acid, acetyl-11-keto-β-boswellic acid from *B. serrata* Roxb. and a diterpenoic cembrene derived alcohol, named “serratol” [7,8]. The first comprehensive study on the essential oil of olibanum of different origins was performed by Obermann in 1977 using the GC-MS technique [9]. In 1985 adetailed review on the “Aden” brand of olibanum was published by Maupetit, who reported 47 new constituents identified in the resinoid and in the oil of olibanum, in addition to 169 formerly identified substances, including the pyrolysis products [10]. Recent studies by Verghese on *B. serrata* oil and by Humprey *et al*. comparing *B. carterii* oil with cumin, ginger and rosemary oil, were essentially reinvestigations of known facts. These studies have highlighted the difficulties in the identification of the origin of olibanum resin, as well as in the determination of standard olibanum oil [11]. Recent studies showed that an ether-soluble gum fraction of 3 to 8 percent oil contains sesquiterpenes, alcohols, esters and boswellic acids. On the other hand, an ether-insoluble gum fraction of 25 to 30 percent contain polysaccarides [12, 13]. Other non-volatile constituents include diterpenoids [14]. The volatile oil contains numerous monoterpenes (in the form of hydrocarbons, alcohols and ketones) and sesequiterpenes, as well as diterpenes. Not unexpectedly, the composition of the oil differs according to the climate and harvest conditions, as well as the geographical source [15]. Numerous boswellic acids have been isolated from the oleo-gum resin derived from Indian, Somalian and Arabian species of *Boswellia* [13,14,15,16,17,18,19]. 

The pharmacological activities of frankincense, as crude extracts, the distilled essential oil and the isolated compounds have been investigated. According to several published reports the frankincense essential oil exhibits *in vitro* antibacterial, antifungal and immunomodulatory activity [15, 20,21,22,23,24,25]. Several other studies investigated the anti-inflammatory, anti-leukotriene, antiacetylcholinesterase, and anticancer activity of the resin and especially its major components, the boswellic acid derivatives [26,27,28,29,30,31,32,33]. 

## Results and Discussion

The yield of volatile oil of Frankincense obtained by hydrodistillation of the finely powdered oleogum resin derived from *Boswellia sacra* was 5.5%. The oil was colourless, with a balsamic slightly spicy and perfumery odor. 

**Figure 1:**
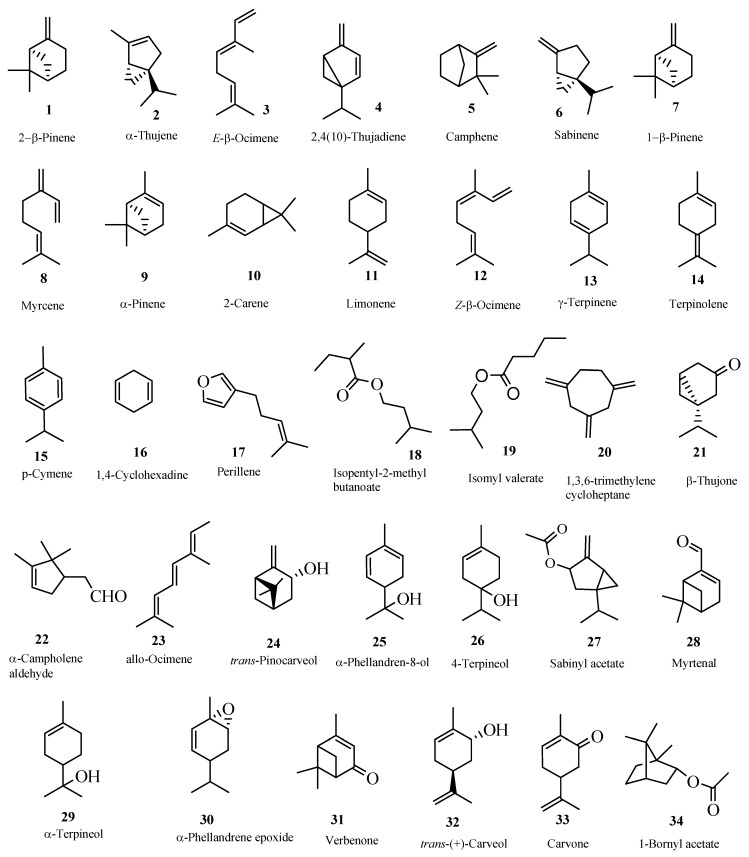
Monoterpenes from the essential oil of the Boswellia *sacra* resin.

**Figure 2:**
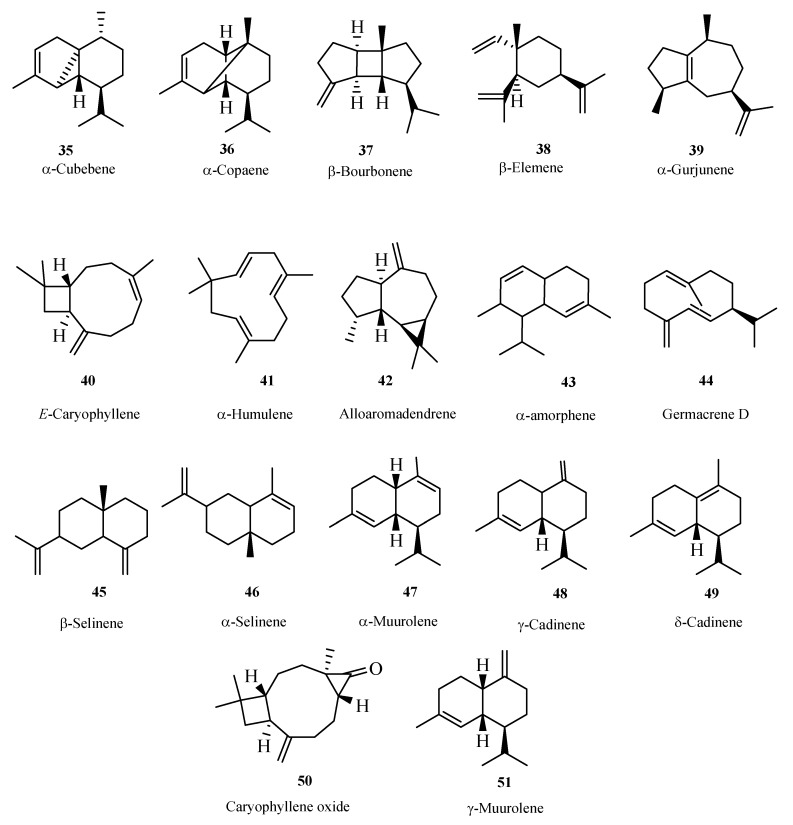
Sesquiterpenes from the essential oil of the *Boswellia sacra* resin.

The GC/MS chromatogram of the hydrodistillate revealed the presence of 34 monoterpenes (Figure 1) and 16 sesquiterpenes (Figure 2) that were identified through comparison of the fragmentation patterns in the resulting mass spectra with those published in literature [34] and using the NIST mass spectral database of the gas chromatograph's computer. 

The oil contains a high proportion of monoterpenes (97.3%) in which *E*-β-ocimene and limonene were the major constituents. The remaining 2.7% was accounted for by sesquiterpenes, in which *E*-caryophyllene was the major constituent. 

The monoterpenes were identified as 2-β-pinene (0.1%), α-thujene (6.6%), *E*-β-ocimene (32.3%), 2,4(10)-thujadiene (0.2%), camphene (0.6%), sabinene (5.2%), 1-β-pinene (1.8%), myrcene (6.9%), α-pinene (5.3%), 2-carene (0.8%), limonene (33.5%), *Z*-β-ocimene (0.2%), γ-terpinene (1.0%), terpinolene (0.4%), *p*-cymene (0.2%), 1,4-cyclohexadiene (0.1%), perillene (0.1%), isopentyl-2-methyl butanoate (0.1%), isomyl valerate (0.1%), 1,3,6-trimethylenecycloheptane (0.1%), β-thujone (0.1%), α-campholene aldehyde (0.2%), *allo*-ocimene (0.1%), *trans*-pinocarveol (0.1%), *p*-mentha-1,5-dien-8-ol (0.2 %), 4-terpineol (0.2%), sabinyl acetate (0.1%), myrtenal (0.1%), α-terpineol (0.1%), α-phellandrene epoxide (0.1%), verbenone (0.1%), *trans*-(+)-carveol (0.1%), carvone (0.1%) and 1-bornyl acetate (0.1%).

The sesquiterpenes were identified to be α-cubebene (0.1%), α-copaene (0.3%), β-bourbonene (0.1%), β-elemene (0.3%), α-gurjunene (0.1%), *E*-caryophyllene (0.9%), α-humulene (0.2%), *allo*-aromadendrene (0.0.1%), α-amorphene (0.1%), germacrene D (0.1%), β-selinene (0.1%), α-selinene (0.1%), α-muurolene (0.1%), γ-cadinene (0.1%), caryophyllene oxide (0.01%) and γ-muurolene (0.1%). 

To the best of our knowledge this represents the first comprehensive GC-MS analysis of *Boswellia sacra* from Oman. It is worth mentioning that there was a brief report on the analysis of *Boswellia sacra* using GLC-MS [35] which reported 22 terpenes, among which α-pinene was the most abundant. We report herein that the essential oil from the botanically certified oleogum resin of *Boswellia sacra* from Oman contains 50 terpenes. 

Unlike the previous analysis [35], which reported the absence of sesquiterpenes in *Boswellia sacra* essential oil; 16 sesquiterpenes were detected in the investigated oil of the oleogum resin of *Boswellia sacra* from Oman. The analysis confirmed the complete absence of the diterpenes in the essential oil of *Boswellia sacra*. 

Surprisingly, the previous analysis has also reported that α-pinene was the most abundant monoterpene In contrast, we found that *E*-β-ocimene (32.3%) and limonene (33.5%) were the predominant compounds and in our analysis α-pinene constituted only 5.3%.

### Comparison with the literature on Boswellia carterii

We compared the results of our analysis for the olibanum sample of certified botanical origin *Boswellia sacra* (Oman) with other spices reported in literature. The triterpene composition of olibanum is well documented in the literature. However, descriptions of the volatile constituents of olibanum species are scarce. Moreover, in almost all the published work, the botanical origin of the olibanum sample was not specified and rather referred to as “purchased from the local market”, so there is confusion in the literature on the chemical composition of olibanum samples regarding the botanical origin of these samples. Purchasing the sample from a local market where the olibanum grows does not prove the sample originated in that region as it could have been conveyed to that country. This kind of confusion can be seen between *Boswellia* *sacra* (Oman) and *Boswellia carterii* (Yemen). This present work on *Boswellia* *sacra* of certified botanical origin illustrated some similarities and differences with those reported in literature. 

The essential oil of *Boswellia carterii* has been one of the most extensively studied olibanum oils. The volatile oil contains a high proportion of esters (*ca.* 40.1%) of which duva-3,9,13-trien-1,5α-diol-1-acetate (21.4%) and octyl acetate (13.4%) are the major components [15]. Within the monoterpenes, the essential oil of *Boswellia* *sacra* was found to contain a low proportion of esters (0.4%). 

Another striking difference between the two species is that in the case of *Boswellia sacra E*-β-ocimene **3** was the major product (32.3%) together with limonene (**11**, 33.5%). The hydrodistillate of *Boswellia carterii* revealed the presence of these two components but in much lower percentages: *E*-β-ocimene (1.7%) and limonene (1.5%) [36]. 

β-Pinene and α-pinene existed in comparable percentages. Mikhaeil *et al*. [15] reported the presence of several sesquiterpenes for *Boswellia carterii* Birdwood. These included α-copaene (0.35%), α-selinene (0.24%), maaliane (0.02%), viridiflorol (0.06%), α-muurolol (0.03%), β-bisabolene (0.15%), *cis*-calamenene (0.01%), spathulenol (0.03%), and *cis*-nerolidol (0.07%). 

Several diterpenes were detected in the oil especially those of the cembranoid skeleton such as, the previously reported cembrene (0.27%), isocembrene (0.28%) [37]. In addition, verticiol (1.22%), duva-4,8,13-trien-1,3α-diol (0.23%), thunbergol (4.07%), duva-3,9,13-trien-1,5α-diol (0.06%), duva-3,9,13-trien-1α-ol-5,8-oxide-1-acetate (0.5%), duva-3,9,13-trien-1,5α-diol-1-acetate (21.35%) were also reported. The analysis of volatile oil of the *Boswellia sacra* resin confirmed the complete absence of diterpenes. 

## Conclusions

The GC/MS analysis of a botanically certified Oleogum Resin of *Boswellia sacra* essential oil revealed the presence of 34 monoterpenes and 16 sesquiterpenes. This comprehensive study should clarify the confusion between the common species of olibanum; the *Boswellia sacra* and *Boswellia* *carterii*. The phytochemical studies of the leaves and bark of the *Boswellia sacra* as well as the biological activities of the resulting essential oils are currently under investigation in our laboratories.

## Experimental

### Sample

The olibanum sample of *Boswellia sacra* (Oman) was collected by Mr. Yaser Al-Rawas from Dhofar Region in Oman. It was taxonomically identified by Dr. Amina Al-Farsi at the Department of Biology, Sultan Qaboos University, Oman.

### Preparation of the volatile oil

The oleogum resin (500 g) was subjected to hydrodistillation using Clevenger’s apparatus until complete exhaustion. The obtained colourless oil was collected, dried over magnesium sulphate and kept at 4 °C until analysis. 

### GC-MS analysis

GC-MS analysis was performed on a Shimadzu model (GC-MS-QP/5050A) instrument. Analytes were separated on a 30 m × 0.32 mm nonpolar capillary column with a phase thickness of 1.0 µm and interfaced with a quadrupole mass spectrometer. The injector and interface temperature were kept at 275 °C and 300 °C respectively and the temperature was programmed from 70 °C to 270 °C at a rate of 3 °C /min. Helium was used as the carrier gas with a linear velocity of 74.6 cm/s and the total flow rate was 39.0 ml/min. The MS operating parameters were: ionization voltage 70 eV, scan rate 500 amu/sec. 
